# Chicory supplementation improves growth performance in juvenile ostriches potentially by attenuating enteritis

**DOI:** 10.3389/fvets.2024.1432269

**Published:** 2024-09-23

**Authors:** Meng Li, Mahmoud M. Abouelfetouh, Eman Salah, Faisal Ayub Kiani, Sha Nan, Mingxing Ding, Yi Ding

**Affiliations:** ^1^College of Veterinary Medicine, Huazhong Agricultural University, Wuhan, China; ^2^Henan Jinlu Special Breeding Farm, Zhengzhou, China; ^3^Department of Surgery, Anesthesiology, and Radiology, Faculty of Veterinary Medicine, Benha University, Moshtohor, Egypt; ^4^Department of Pharmacology, Faculty of Veterinary Medicine, Benha University, Moshtohor, Egypt; ^5^Department of Clinical Sciences, Faculty of Veterinary Medicine, Bahauddin Zakariyah University, Multan, Pakistan

**Keywords:** chicory, chicoric acid, intestinal inflammation, mortality rate, ostriches, microbiota

## Abstract

**Introduction:**

Enteritis and dysbiosis are the major causes of high morbidity and mortality of juvenile ostriches. Chicory (CC) has been proven to have excellent antioxidant, anti-inflammatory, and antibacterial activities. However, it’s unclear whether CC could improve the survival rate of juvenile ostriches by relieving enteritis and correcting dysbiosis.

**Materials and methods:**

South African ostrich hatchlings (*Struthio camelus domesticus*) were fed with and without a CC-supplemented diet, and the body weight gain and mortality were compared over 4 months of age. Fresh fecal samples of clinically healthy ostriches were collected, and 16S DNAs were analyzed. Moreover, ostrich chicks with LPS-induced enteritis were fed with different dosages (0, 20, 40, and 80 mg/kg) of chicoric acid (CA), a major bioactive component of CC, for five consecutive days. The expression levels of tight junction (TJ)-related proteins and inflammatory mediators in the ilea were detected with western blot and immunofluorescence.

**Results:**

The ostrich chicks fed on the CC-supplemented diet began to increase in weight at the 1st month of age and became remarkably heavier at the fourth month (*p* < 0.01) compared with those fed on the non-CC-supplemented diet. Additionally, the mortality percentage was lower in the chicks fed on the CC-supplemented diet than those fed on the non-CC-supplemented diet (19% vs. 36%, respectively). The diet with the CC supplementation significantly increased the abundance of *Phascolactobacteria* (linear discriminant analysis; LDA >4) and *Bacteroidota* (26.7% vs. 17.7%, respectively) as well as decreased the enrichment of *Clostridium* (5.0% vs. 9.1%, respectively) in the ostrich ilea compared to the diet without CC. The supplementation of CA at a dose of 80 mg/kg significantly increased the expression level of ZO-1 and claudin-3 (*p* < 0.0001) and suppressed the levels of IL-1β, IL-6, and TNF-α (*p* < 0.0001) in ostriches with LPS-induced ileitis.

**Conclusion:**

Our results substantiate that CC or CA supplementation in a diet could effectively improve growth performance and reduce mortality in juvenile ostriches via modulating the gut microbiota and attenuating enteritis.

## 1 Introduction

The ostrich industry is gaining much attention worldwide due to the high market value of its products and byproducts (1). However, the high prevalence of juvenile mortality has a negative influence on ostrich farming as well as the breeders’ enthusiasm (2). Ostrich hatchlings appear to have structural and functional deficiencies in the intestinal mucosa that lead to inadequacy in the digestive function (3). Additionally, the inherent coprophagy behavior of ostriches contributes to a high incidence of intestinal ailments and mortality in ostrich chicks (4, 5). The mortality rate in the young population (up to 3 months of age) is reported to be 30–40%, primarily due to intestinal tract diseases and/or an imbalance of the gut microbiota (dysbiosis) (2, 6). Dysbiosis is characterized by a reduction of beneficial bacteria and an overgrowth of pathogenic bacteria (2). Bacterial enteritis has been recognized as the main cause of mortality in ostrich chicks (7). The diversity of microbes and disease response vary markedly across the length of the intestinal tract. Among gut regions, the ileum is more sensitive to enterocolitis in ostriches, showing a higher degree of inflammation and conspicuous evidence of dysbiosis (2). A vast variety of antimicrobial agents were extensively used to prevent and/or treat bacterial enteritis in the poultry industry. These communal practices result in the emergence of drug-resistant pathogens, gut dysbiosis, delayed growth, and increased drug residues in animal products with a potential public health hazard (8–10). The discovery of new therapeutic agents and the evolution of biologically active substances as safe substitutes have received significant attention in this regard (11–13). Herbal products offer a vast resource of new medicinal agents for the protection and/or remedy of various diseases in various animal species (14–16). Therefore, it’s important to look for alternatives that could attenuate enteritis in ostrich chicks, improve gut health and growth performance, and limit the use of antimicrobials.

Chicory (CC) (*Cichorium intybus*), a highly palatable perennial plant grown worldwide, is well-known for its numerous nutritional and medicinal properties (17). Due to its versatile bioactive ingredients, CC could be used in livestock production as a sustainable, beneficial forage-based nutrient (18). Various *in-vivo* trials have demonstrated intriguing consequences regarding CC supplementation in various livestock. CC could amend the gut microbiota and improve digestibility and development of the gastrointestinal tract in growing pigs (19, 20) and sheep (21). Dietary inclusion of CC in a forage-dependent diet is helpful to enhance feed intake, feed conversion efficiency, and growth performance in beef steers (22). In addition, CC has been reported to modify intestinal structure and enhance gut microbial diversity in broiler chickens (23). Supplementation of CC for broilers could also enhance growth performance with an improved immune status and blood lipid profile, such as cholesterol, triglycerides, and low-density lipoprotein levels (24). Chicoric acid (CA), a major biological constituent of CC, is considered a bioactive component with potent anti-oxidant, anti-inflammatory, and anti-bacterial bioactivities (17, 25, 26). CA has been reported to effectively lessen inflammation and oxidative stress induced by lipopolysaccharide (LPS) in acute liver injury (27), acute lung injury (28), and BV-2 microglia cell models (25). However, the beneficial effect of CC or CA on the enteritis of juvenile ostriches has not yet been investigated.

Up to date, CC supplements have not yet been extensively used in ostrich production. The objective of this current study was to elucidate the influence of CC on growth performance and mortality rate associated with enteritis in juvenile ostriches. We hypothesized that CC supplementation could improve growth performance, attenuate enteritis, and decrease the incidence of mortality by modulating the gut microbiota and relieving intestinal inflammation.

## 2 Materials and methods

### 2.1 Animals

South African black-necked ostrich hatchlings (*Struthio camelus domesticus*) at Henan Jinlu Special Breeding Co., Ltd., (Zhengzhou, Henan Province, China) were selected for the present study. The ostrich hatchlings were kept in a warm, dry, and draft-free environment with sufficient space to move around and allowed to have free access to water and food. The brooding temperature (BT) of the hatchlings was set at 33.5°C during the 1st week of age and then gradually decreased to reach 25°C at the end of the 4th week of age. The BT was further reduced to 20°C at the end of the 8th week, then adjusted to 18°C from the 9th week to the end of the experiment. Dehumidifiers were used to reduce the ambient moisture during brooding. The level of humidity was between 25 and 55% during the entire period of the experiment. This study was approved by the Animal Protection and Utilization Committee of Huazhong Agricultural University (ID number: HZAUBI-2023-0003).

### 2.2 Study design

#### 2.2.1 Effect of chicory forage on enteritis and mortality in juvenile ostriches

To determine the effect of CC forage on growth performance, 200 zero-day-old healthy ostrich hatchlings with weights (828.9 ± 24.2 grams) from a newly hatched batch were enrolled in this prospective randomized design. All experimental hatchlings were initially allocated into five allocations (A, B, C, D, and E) (*n* = 40 each). These allocations were selected in a random order to assign two equal-sized groups (non-chicory control (NC) and chicory group (CC); *n* = 100) using computer software.^1^ The chicks were kept in an outdoor enclosure (9 × 30 m) with soil substrate during the daytime and in an indoor pen of approximately 3.5 × 4 m during the night. Both NC and CC chicks were fed concentrate and green forage at a consistent weight ratio of 1:1. The green forage in the NC group was composed of amaranth, cabbage, and *Ipomoea aquatica*, which was replaced by CC fodder in the CC group. The nutritional composition of the supplied concentrate is shown in Table 1. The chicks were allowed *ad libitum* access to water. Body weights were recorded monthly in both groups up to 4 months of age. The chicks’ clinical manifestations were monitored daily, and the deceased chicks were autopsied for gross and histological examination. The mortalities with enteritis in both the NC and CC groups were calculated.

**Table 1:** Nutritional composition of the supplied concentrate.

Component	Value	Component	Value	Component	Value
Metabolizable energy (MJ/kg)	11.3 MJ	Vit A	12,000 IU	Vit B12	0.1 mg
Crude protein (%)	21	Vit D	3,000 IU	Pantothenic acid	18 mg
L-Lysine (%)	1.1	Vit E	30 mg	Niacin	80 mg
Methionine (%)	0.45	Vit K	3 mg	Biotin	0.3 mg
Ca (%)	1.5	Vit B1	4 mg	Folic acid	2 mg
Available P (%)	0.75	Vit B2	12 mg	Choline	500 mg
NaCl (%)	0.25	Vit B6	8 mg	Fe	160 mg
Cu	20 mg	Mn	120 mg	I	0.6 mg
Se	0.3 mg	Zn	80 mg	Co	0.5 mg
Corn (g)	598	Soybean (g)	270	Fish meal	15 g

All elements were supplied per kilogram concentrate and provided in accordance with China Ostrich Breeding and Development Association.

#### 2.2.2 Gut microbiota analysis

To investigate the effect of CC on the gut microbial community, we collected six fresh fecal samples of one-month-old clinically healthy ostriches from each of the CC and NC groups in the experiment above. The total microbial DNA was extracted using the OMEGA Mag-Bind Soil DNA Kit (M5635-02) (Omega Bio-Tek, Norcross, GA, United States) according to the manufacturer’s instructions. The quantity of the extracted DNA was determined using an ultraviolet-visible spectrophotometer (NanoDrop 2000, United States), and its integrity was assessed using agarose gel electrophoresis. DNA samples were uniformly diluted to 20 ng/μL. The DNA of the 16S V3V4 region was PCR amplified using specific primers (341F, 5′-CCTAYGGGRBGCASCAG-3′; and 806R, 5′-GGACTACNNGGGTATCTAAT-3′). The PCR amplification conditions were as follows: initial denaturation at 98°C for 2 min, denaturation at 98°C for 15 s, annealing at 55°C for 30 s, extension at 72°C for 30 s, final extension at 72°C for 5 min, 10°C hold, 25–30 cycles. PCR amplification products were subjected to fluorescent quantification using the Quant-iT PicoGreen dsDNA Assay (Thermo Fisher Scientific Inc., China). According to the fluorescent quantification results, the samples were mixed in the appropriate proportions to meet the sequencing requirements. The microbial DNA was processed to construct metagenome sequencing libraries using TruSeq Nano DNA LT Library Preparation Kit (Illumina, Inc. CA, United States). The library DNA fragments were PCR amplified to enrich the sequencing library template. The amplified products were then purified using AMPure XP system (Beckman, Coulter, Beverly, CA, United States). The enriched library was further purified, and the final fragment selection was performed using 2% agarose gel electrophoresis. Before sequencing, the library was subjected to quality control using an Agilent Bioanalyzer system with the Agilent High Sensitivity DNA Kit (Agilent, CA, United States). Sequencing was performed on the MiSeq platform using 2 × 300 bp paired-end reads with the MiSeq Reagent Kit V3 (600 cycles) (Illumina, Inc. CA, United States). To ensure high sequencing quality, the optimal sequencing length for target fragments was between 200–450 bp. Sequences were denoised or clustered into Operational Taxonomic Units (OTUs) using the QIIME2 (2019.4) DADA2 software. Based on the distribution of ASVs/OTUs in different samples, the α diversity level of each sample was assessed. Various unsupervised ordination, clustering methods, and statistical tests were employed to measure and determine the significance of β diversity differences and assess the differences in species abundance between samples. Based on the bacterial metagenomic data, the predicted functional pathways of the bacterial communities were extracted from the Kyoto Encyclopedia of Genes and Genomes (KEGG) database.

#### 2.2.3 Effect of chicoric acid on intestinal inflammation induced by LPS in juvenile ostriches

To determine whether CC could relieve enteritis via its bioactive component CA, clinically healthy 42 ten-day-old South African ostrich chicks (1130.83 ± 34.3 grams) were selected and randomly allocated into seven groups (*n* = 6 each): non-treatment control (NC), CA control (CAC, 80 mg/kg of CA was supplemented in diet), LPS + CA (LC, 1.5 mg/kg LPS intraperitoneally injected, and 0, 20, 40, and 80 mg/kg of CA were supplemented in diet), and LPS + amoxicillin (LA, 1.5 mg/kg LPS intraperitoneally injected, and 20 mg/kg of amoxicillin was supplemented in diet). LPS (Sigma Aldrich, Germany) was administered on day 0 of the experiment. The ostrich chicks were fed with the same ratio of concentrate and green forage (amaranth, cabbage, and *Ipomoea aquatica*) as in the above experiment. CA (Shanxi Qinling Biological Engineering Company, China) or amoxicillin (CSPC Pharmaceutical Group Limited, China) were supplemented in the diet, starting at day 0, for five consecutive days. The chicks were weighed on days 1, 3, and 5. On day 5, the chicks were anesthetized with Zoletil 50 (0.1 mg/kg; IM; Virbac, France), and the ilea were harvested. The ileal samples were then divided into two parts: one stored at −80°C for protein analysis, and the other was fixed in a 10% neutral formalin solution for histopathological examination.

### 2.3 Histopathological examination

The harvested ileal samples were fixed in 10% neutral-buffered formalin, dehydrated in serial dilutions of ethanol concentrations ranging from 50 to 100%, cleared in xylene, and embedded in paraffin. The paraffin-embedded tissues were cut into 5 μm thick sections. The intact slices were chosen for conventional H&E staining protocol. For staining, the tissue sections were deparaffinized in three xylene baths, and hydrated by passing through decreasing concentrations of alcohols (100, 95, 80, 75%) and water. The tissue was stained with hematoxylin for three to 5 min, followed by a water wash for 5 min. Then, the tissue was dipped in 1% acid alcohol for few seconds, followed by ammonia water bathing. After a tap water wash, the tissue slices were stained with 1% eosin for one to 2 min. Next, the sections were dehydrated in increasing concentrations of alcohol baths and cleared in two xylene baths. Finally, the slides were mounted and examined by a professional pathologist under a Leica DM4B light microscope (Leica Microsystems, Germany). Tissue evaluation was based on a modified histological scoring [none (0), mild (1), moderate (2), and severe (3)] (29), describing the severity and degrees of villous inflammation, cellular infiltration, congestion, and epithelial cell shedding. Additionally, the average villus heights of fifteen well-oriented villi with intact lamina propria were measured from the crypt opening to the end of the villi (30) using Aperio ImageScope software x64 (Leica Biosystem Imaging, Inc., Germany).

### 2.4 Western blot

Protein extraction from the ileum tissue was performed using RIPA Lysate Buffer (Merck Ltd., Darmstadt, Germany), and the concentrations were measured by the BCA Protein Assay Kit in accordance with the manufacturer’s procedure, and then diluted to the same concentration. Proteins were separated by SDS-PAGE and transferred to a PVDF membrane with a Trans-Blot^®^ TurboTM transfer system (Bio-Rad, Hercules, CA, United States). Then, the membrane was blocked with 5% skim milk in Tris-buffered saline (TBS) at 37°C for 2 h, and immunolabeled with TBS-diluted primary antibodies at 4°C overnight. Primary antibodies used in this study were rabbit polyclonal anti-TLR4 (1:1,000 dilution) (ABclonal, Inc., Wuhan, China), rabbit polyclonal anti-P-P65 (1:1,000 dilution) (Cell Signaling Technology, Inc., United States), rabbit polyclonal P65 (1:1,000 dilution) (AbMart Pharmaceutical Technology Co., Ltd., Shanghai, China), rabbit polyclonal anti-IL-1β (1:1,000 dilution), rabbit polyclonal anti-IL-6 (1:1,000 dilution) (ABclonal, Inc., Wuhan, China), anti-TNF-α (1:1,000 dilution) (Boaosen Biotechnology Co., Ltd., Beijing, China), rabbit polyclonal anti-ZO-1 (1:1,000 dilution), anti-claudin-3 (1:1,000 dilution) (Wanlei Biotechnology Co., Ltd., Shenyang, China) and rabbit polyclonal anti-GAPDH (1:10,000 dilution) (ABclonal, Inc., Wuhan, China). Following TBST wash for 3 × 5 min, the blots were then incubated together with horseradish peroxidase (HRP)-conjugated donkey anti-rabbit (1:50,000 dilution) (Servicebio Technology Co., Ltd., Wuhan, China) as secondary antibodies at 37°C for 1 h. The antigen-antibody complex was visualized by a Clarity Max^™^ Western ECL Substrate (Bio-Rad) and the imaging of immunoreactive proteins was taken using the Bio-Rad ChemiDoc^™^ MP imaging system. The protein bands were scrutinized by Image J 1.46r (National Institutes of Health, United States). The target protein values were denoted as the proportion of the optical density of the protein bands in treatment groups to the density of the corresponding control band in the negative control group.

### 2.5 Immunofluorescence of ZO-1 and claudin-3 proteins

To determine the effect of CA on the intestinal barrier of ostrich chicks with enteritis, the main TJ proteins were measured with immunofluorescence. The harvested ilea were fixed in 10% neutral-buffered formalin, dehydrated in serial dilutions of ethanol concentrations (50–100%), cleared in xylene, and embedded in paraffin. The paraffin-embedded tissues were sectioned into 5 μm-thick sections. The sections were treated with 5% BSA at room temperature for 30 min before being incubated with the primary antibody at 4°C for 12 h. The sections were washed with PBS, incubated with the secondary antibody at room temperature for 30 min, and stained with 4,6-diamidino-2-phenylindole (DAPI; 1:5,000, Beyotime, SH, China) for 5 min at room temperature. Primary antibodies were rabbit polyclonal anti-ZO-1 (1:500 dilution) and anti-claudin-3 (1:500 dilution) (Wanlei Biotechnology Co., Ltd., Shenyang, China), and secondary antibodies were HRP-conjugated Cy3 labeled goat anti-rabbit (1:500), and FITC-conjugated goat anti-rabbit antibodies (1:500 dilution) (Servicebio Technology Co., Ltd., Wuhan, China). The stained sections were examined using flourescence microscope (Olympus cellsens Entry, Japan). The fluorescence signal was analyzed using Image J 1.46r (National Institutes of Health, United States).

### 2.6 Statistical analysis

Sequencing data analysis was performed using QIIME2 (v2.0) software. The DADA2 method was employed to filter, denoise, merge, and remove chimeric and singleton sequences from the original data. ASVs (equivalent to representative operational taxonomic units (OTU) sequences) were obtained through 100% similarity clustering. Species annotation, taxonomic composition, α diversity, and β diversity analyses were conducted using custom scripts in QIIME2 and the R programming language. Corresponding charts were generated to visualize the results. The assumption of normality in α diversity indices was tested using the Kolmogorov–Smirnov (KS) analysis. The variables of the α diversity did not meet the basic assumption and were therefore analyzed by non-parametric tests (31). Kruskal–Wallis rank sum test with post-hoc Dunn’s tests were used to compare α diversity indices (Chao 1, Good’s coverage, Shannon, and Pielou) and the data were expressed as median (IQR). Principal coordinates analysis (PCoA) was performed in R using scripts to output the PCoA coordinates of sample points and plot them into a two-dimensional scatter plot. The discrimination of distance metrics between groups was based on the ordination method and verified to ensure consistency (31, 32). Permutational multivariate analysis of variance (PERMANOVA) was used to analyze Bray–Curtis and weighted UniFrac distances to confirm the dissimilarity. LEfSe [linear discriminant analysis (LDA) effect size] analysis using Kruskal–Wallis and Wilcoxon rank sum tests was used to analyze the differences in the microbial community compositions. LDA score >4 was considered statistically significant. Phylogenetic Investigation of Communities by Reconstruction of Unobserved States (PICRUSt2) method was used to predict microbial metabolic functions. Abundance values for metabolic pathways based on the metabolic pathway KEGG database were calculated. Weight gain, mortality, and numerical data of western blot and immunofluorescence were compared using one-way ANOVA analysis with least significance difference (LSD) using SPSS software (IBM statistics 26). Data are presented as mean ± SD. A statistical significance was defined as *p*-value <0.05.

## 3 Results

### 3.1 Chicory forage improved growth and reduced enteritis-associated mortality in juvenile ostriches

**Figure 1:**
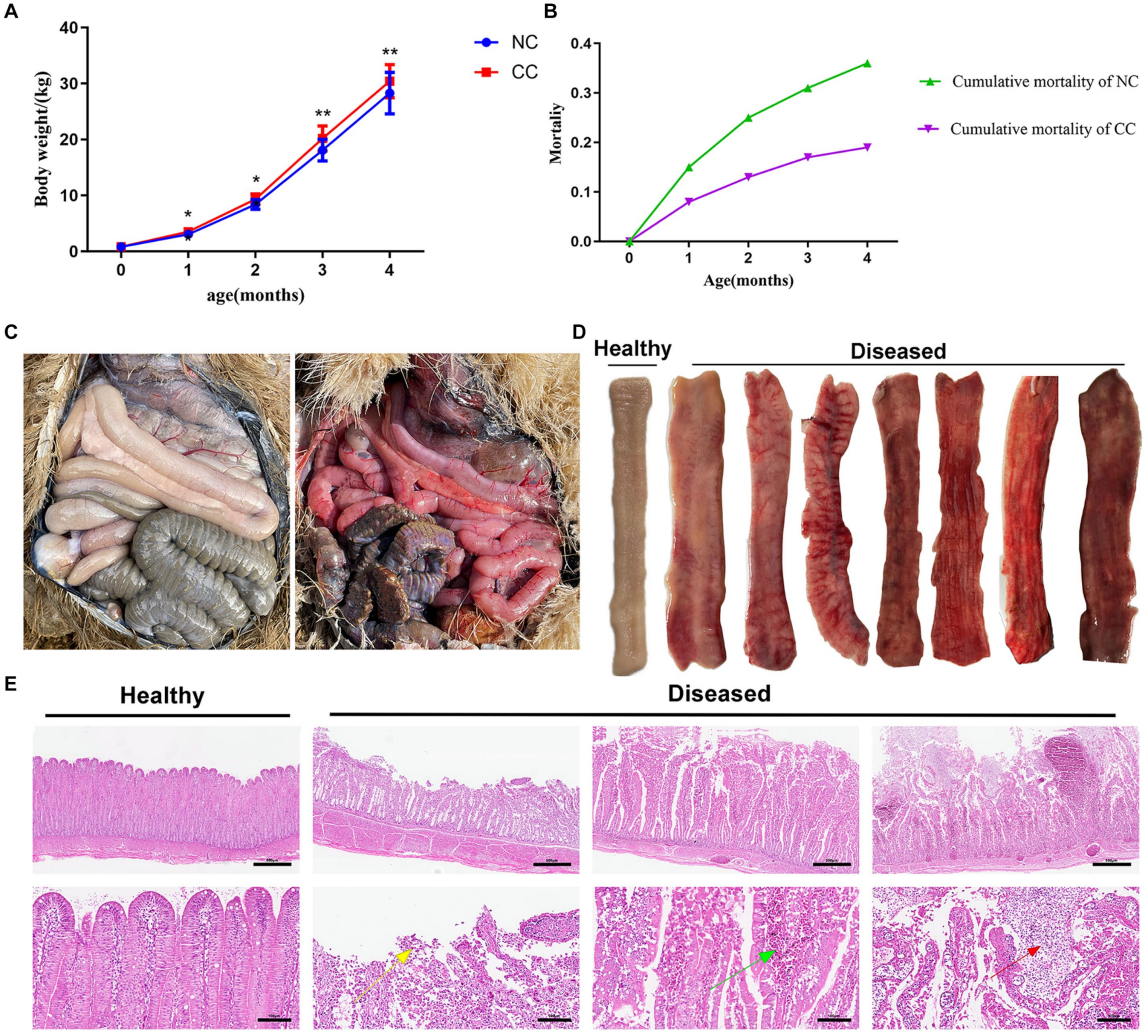
Effect of chicory supplementation on body weight and mortalities due to intestinal inflammation in ostrich chicks. **(A)** Weight changes in the non-chicory group (NC; *n* = 100) and chicory group (CC; *n* = 100) during the experiment. **(B)** Cumulative mortality in the ostriches throughout the entire period of the experiment (4 months). **(C)** Left: guts of clinically healthy ostriches; right: generalized gut inflammation in deceased ostriches. **(D)** Gross appearance of ileal mucosa in healthy and diseased ostriches with enteritis. **(E)** Representative H&E histopathological examination of the ilea of healthy ostriches and diseased ostriches with enteritis shows the shedding of villous epithelial cells (yellow arrow), congestion in the intestinal villi (green arrow), and substantial lymphocytic infiltration (red arrow). Image magnification is 40× and 200× and scale bar = 500 and 100 μm. The asterisk symbol * indicates *p* < 0.05; ** indicates *p* < 0.01.

The CC-supplemented chicks showed an increase in weight compared to the NC chicks (*p* < 0.05) at day 30 to 120 of the experiment (Figure 1A). There was a death proportion of ostriches in both CC and NC, but the mortality percentage in the NC group was far greater than that in the CC group at day 120 (36% vs. 19%; Figure 1B). The autopsy showed that enteritis contributes to 25% of the mortality in the NC group and 9% in the CC group. In both groups, post-mortem inspection of the deceased chicks with enteritis revealed an inflamed red gut characterized by a varying degree of congestion, bleeding, abnormal coloration, and thinning of the intestinal wall (Figure 1C right; Figure 1D) compared to those in the clinically healthy ostrich chicks (Figure 1C left; Figure 1D). Histologically, the deceased chicks with enteritis showed a loose arrangement of small intestinal villi, accompanied by necrosis and the shedding of villous epithelial cells. As well, the ileal villi showed a varying degree of congestion with substantial lymphocytic infiltration (Figure 1E).

### 3.2 Chicory forage modified gut microbiota of juvenile ostriches

**Figure 2:**
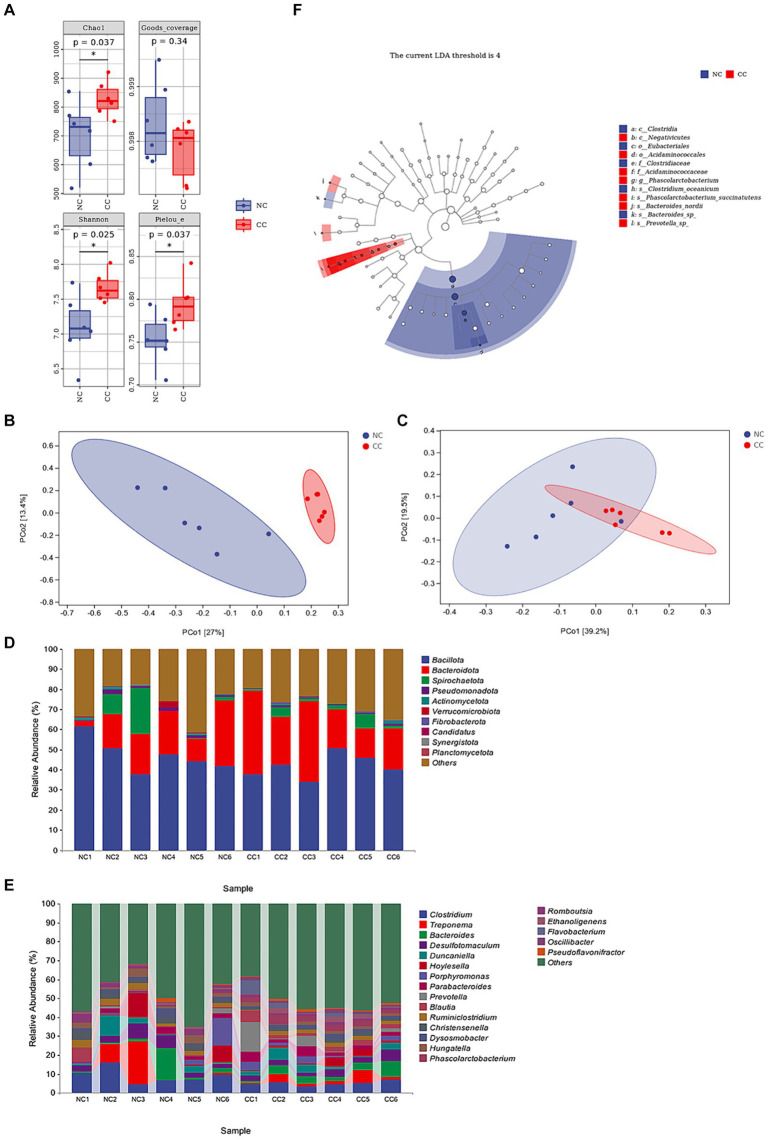
Effect of chicory supplementation on gut microbiota screened from fecal specimens of non-chicory group (NC, *n* = 6) and chicory group (CC, *n* = 6) in ostriches. **(A)** Alpha diversity (Chao 1, Good’s coverage, Shannon, and Pielou indices) in both NC and CC groups; **(B,C)** Principal coordinates analysis (PCoA) plots of Bray–Curtis distance and weighted UniFrac distance between the two groups. Ellipses denote 95% confidence intervals. **(D)** Abundance of the fecal microbiota at the phylum level (top 10) in samples of both groups. **(E)** Abundance of intestinal microbiota at the genus level (top 20). **(F)** LEfSe analysis integrated with LDA scores revealed differential biomarkers, LDA scores >4 was considered statistically significant. The symbol * indicates *p* < 0.05.

A total of 770 common operational taxonomic units (OTUs) was identified between the NC and CC groups, representing approximately 17.3% of the total OTUs. The rarefaction curves and species accumulation curves in both groups revealed that almost all bacteria in the fecal samples were detected. Furthermore, the rank abundance curves showed a commendable level of uniformity and ASV/OTUs abundance in the samples (Supplementary Figure S1). Good’s coverage ranged from 99.71 to 99.95% in the NC and CC groups (*p* = 0.34), indicating the vast majority of bacteria was recognized. The CC group exhibited a significant increase in Chao1 (829.347 ± 61.452; *p* = 0.037) and Shannon indices (0.985 ± 0.006; *p* = 0.025) as compared to the NC group (701.918 ± 121.335 and 0.975 ± 0.011 respectively). The Chao1 and Shannon indices of the CC group indicated a higher species richness compared with the NC group. Additionally, Pielou’s evenness in the CC group was significantly higher compared with the NC group (0.794 ± 0.028 vs. 0.754 ± 0.030, respectively) (*p* = 0.037) (Figure 2A). Based on Bray–Curtis and weighted UniFrac distance methods, PCoA plots demonstrated a substantial distance between the NC and CC groups on the coordinate axes. The discrimination between the groups was distinct, and the intra-variability between the samples in the NC group is much lower than the intra-variability between the samples in the CC group, indicating changes in the composition and structure of the gut microbiota between the two groups (Figures 2B,C). The pairwise PERMANOVA displayed a statistically significant difference between the distance metrics observed for the NC group and the CC group (*p* < 0.005).

The top species at the phylum level of the samples of both the NC and CC groups are shown in Figure 2D, with *Bacillota*, *Bacteroidota*, and *Spirochaetota* constituting the major phylum and making up more than 70% of the total. The average abundances of these phyla in the NC and CC groups were 47.2, and 41.7% for *Bacillota*, 17.7, and 26.7% for *Bacteroidota*, and 5.7, and 2.8% for *Spirochaetota*, respectively. The top species at the genus level were displayed in Figure 2E, the relative abundances for *Clostridium* were 9.1, 5.0%, *Treponema* were 5.6, 2.8%, *Bacteroides* were 3.8, 4.1%, *Desulfotomaculum* were 4.4, 3.3%, *Duncaniella* were 3.3, 3.2%, in the NC and CC groups, respectively. LEfSe analysis (LDA score >4) demonstrated differential microbiota-based biomarkers with higher abundances of *Christensenellaceae*, *Clostridium*, and *Christensenella* found in the NC group, and *Acidaminococcales*, *Acidaminococcaceae*, *Phascolarctobacterium*, and *Oscillibacter* in the CC group (Figure 2F). Four species at the genus level, two upregulated and two downregulated in each group, are depicted in Supplementary Figure S2, which provides a thorough explanation of these differential variations. On the basis of KEGG pathway analysis, the predicted metabolic pathways for microbial communities were significantly influenced by the supplementation of CC. The metabolic pathways of amino acid biosynthesis and nucleoside and nucleotide biosynthesis were significantly increased in the CC group compared to the NC group (*p* = 0.034; *p* = 0.013, respectively). However, the CC group showed a decreased level of fatty acid and lipid biosynthesis pathway compared to the NC group (*p* = 0.019) (Supplementary Figure S3).

### 3.3 Chicoric acid alleviated LPS-induced intestinal inflammation in juvenile ostriches

**Figure 3:**
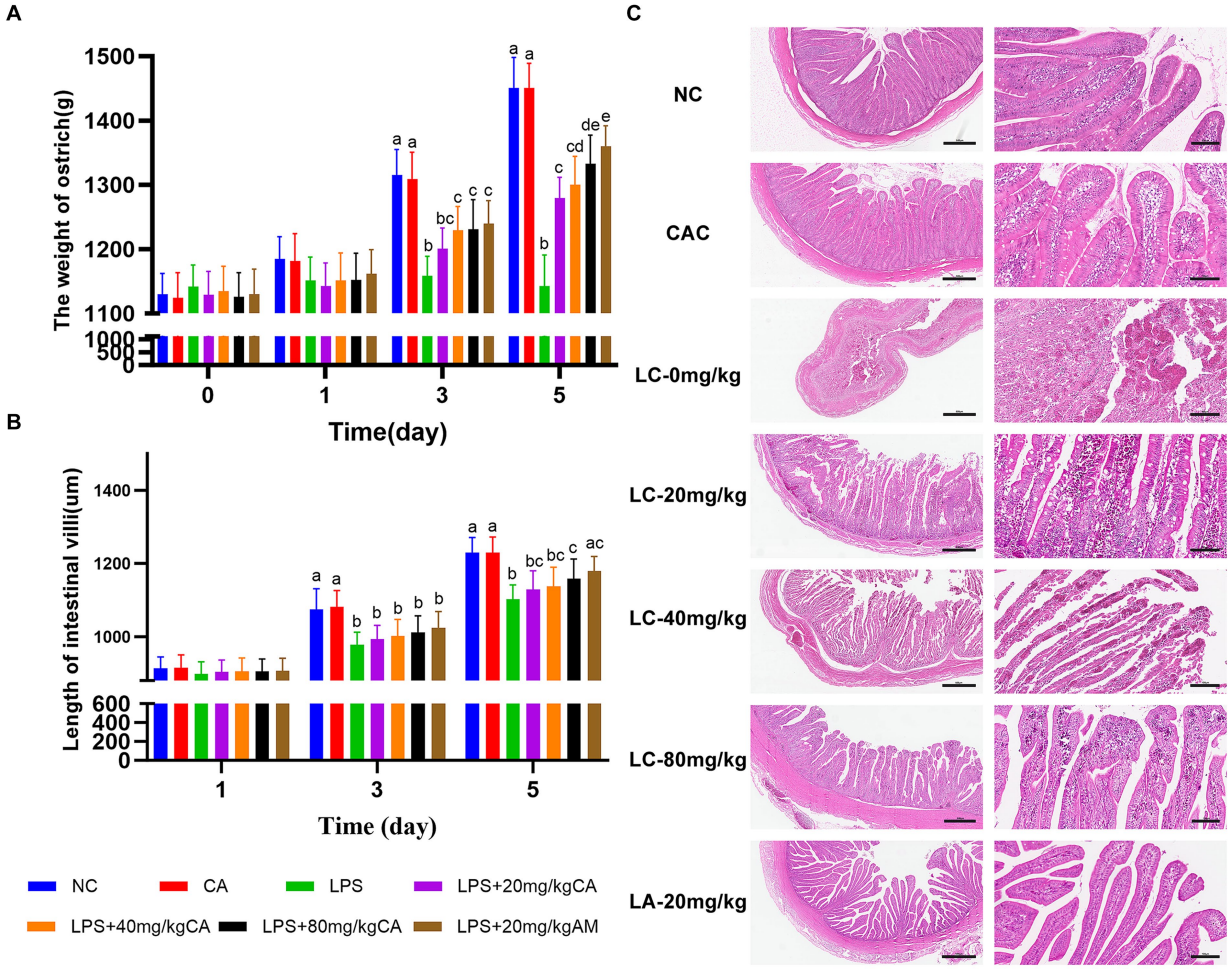
Effect of chicoric acid (CA) on body weight and ilea histology of lipopolysaccharide (LPS)-challenged ostriches. **(A)** Body weight of ostrich chicks (g) on days 1, 3, and 5. **(B)** Length of ileal villi (μm) on days 1, 3, and 5. **(C)** H&E microscopic pictures of ilea of ostrich chicks on day 5 after being challenged with LPS and fed with CA (20, 40, and 80 mg/kg) and amoxicillin (AM; 20 mg/kg) (*n* = 6). NC, non-CA supplementation; CAC, 80 mg/kg CA supplementation; LC-0 mg/kg, LC-20 mg/kg, LC-40 mg/kg and LC-80 mg/kg indicate LPS 1.5 mg/kg, IP, and 0, 20, 40, and 80 mg/kg CA were supplemented in the diet, respectively, and LA-20 mg/kg indicates LPS 1.5 mg/kg, IP, and 20 mg/kg AM was supplemented in the diet. Data were presented as mean ± SD. Different superscript letters indicate a significant difference (*p* < 0.05). H&E image magnification is 40× and 200× and scale bar = 500 and 100 μm.

Chicks treated with LC-0 mg/kg exhibited a decrease in body weight compared with those in the NC and CAC groups on day 3 and 5 (*p* < 0.0001). Chicks received LC-(40 and 80) mg/kg and LA-20 mg/kg became heavier on day 3 and their weight gain was remarkably increased on day 5 as compared to those given LC-0 mg/kg (*p* < 0.05). The LC-80 and LA-20 mg/kg treatments showed non-significant difference in body weight during the entire period of the experiment (Figure 3A).

Both the LC-80 and LA-20 mg/kg treatments showed a significant increase (*p* = 0.043; *p* = 0.007, respectively) in ileal villus length on day 5 compared with the LC-0 mg/kg treatment (Figure 3B). Histologically, the LC-0 mg/kg treatment caused moderate to severe epithelial cell shedding, lymphocyte infiltration, and bleeding on day 5. There was no pathological change observed in the villous morphology of ostrich chicks supplemented with LC-80 and LA-20 mg/kg, while the ilea of ostrich chicks treated with LC-(20 and 40) mg/kg showed mild hemorrhagic congestion in the villi infiltrated with lymphocytes at day 5 (Figure 3C).

### 3.4 Chicoric acid downregulated expression levels of TLR4/NF-κBp65 pathway-related proteins and upregulated TJ-related proteins

**Figure 4:**
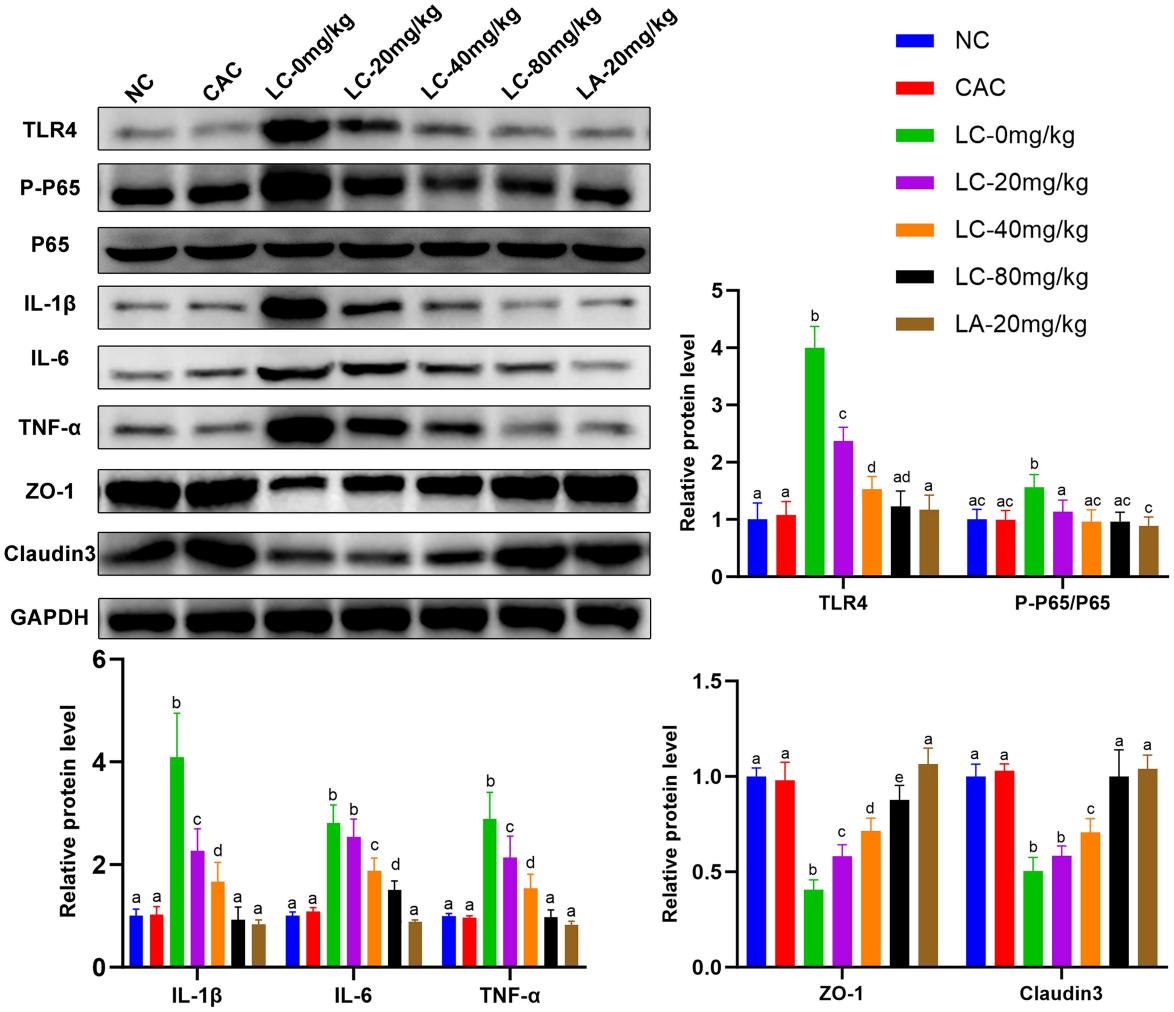
Effect of CA on expression levels of TLR4/NF-κBp65 pathway-related proteins and tight junction (TJ) proteins in the ileal tissues of lipopolysaccharide (LPS)-challenged ostrich chicks on day 5 after being challenged with LPS and fed with CA (20, 40, and 80 mg/kg) and amoxicillin (AM; 20 mg/kg) (*n* = 6). NC, non-CA supplementation; CAC, 80 mg/kg CA supplementation; LC-0 mg/kg, LC-20 mg/kg, LC-40 mg/kg and LC-80 mg/kg indicate LPS 1.5 mg/kg, IP, and 0, 20, 40, and 80 mg/kg CA were supplemented in the diet, respectively, and LA-20 mg/kg indicates LPS 1.5 mg/kg, IP, and 20 mg/kg AM was supplemented in the diet. Data were presented as mean ± SD. Different superscript letters indicate a significant difference (*p* < 0.05).

The effect of CA treatment on the intestinal inflammation in ostrich chicks was evaluated by measuring the expression biomarkers of toll-like receptor 4 (TLR4)-dependent nuclear factor kappa B (TLR4/NF-κB) pathway-related proteins and TJ-related proteins on day 5 (Figure 4). The levels of TLR4, P-P65, IL-1β, IL-6, and TNF-α were increased (*p* < 0.0001) in the LC-0 mg/kg group compared with the NC and CAC groups. However, the levels of these inflammatory factors were reduced (*p* < 0.05) in chicks receiving LC-(20, 40, and 80 mg/kg) and LA-20 mg/kg compared with those treated with LC-0 mg/kg. The LC-80 mg/kg caused a significant decrease (*p* < 0.001) in IL-1β, IL-6, and TNF-α compared with the LC-20 mg/kg and LC-40 mg/kg. There was no difference in the levels of TLR4, P-P65, IL-1β, and TNF-α between the LC-80 mg/kg treatment and the NC or CAC group.

Chicks treated with LC-0 mg/kg resulted in a significant decrease in the levels of ZO-1 and claudin-3 proteins compared with the NC and CA groups (*p* < 0.0001). These proteins in the LC-(20, 40, and 80) mg/kg and LA-20 mg/kg-treated chicks were upregulated compared with those administered LC-0 mg/kg (*p* < 0.05). There was no difference in the levels of claudin-3 between LC-80 and LA-20 mg/kg treatments.

**Figure 5:**
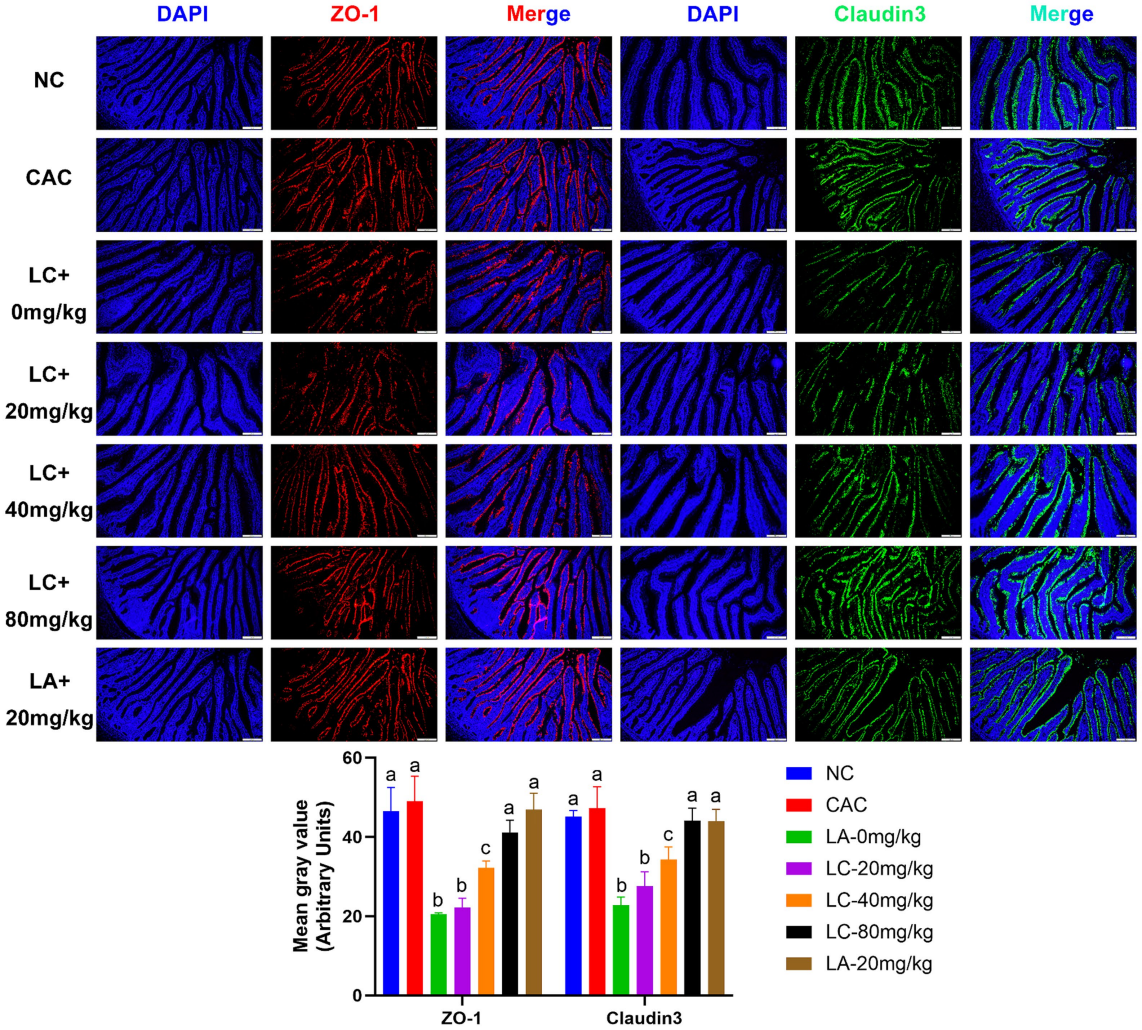
Effect of chicoric acid (CA) on immunofluorescence density of tight junctions (TJs) in the ileal tissues of lipopolysaccharide (LPS)-challenged ostrich chicks on day 5 after being challenged with LPS and fed with CA (20, 40, and 80 mg/kg) and amoxicillin (AM; 20 mg/kg) (*n* = 6). NC, non-CA supplementation; CAC, 80 mg/kg CA supplementation; LC-0 mg/kg, LC-20 mg/kg, LC-40 mg/kg and LC-80 mg/kg indicate LPS 1.5 mg/kg, IP, and 0, 20, 40, and 80 mg/kg CA were supplemented in the diet, respectively, and LA-20 mg/kg indicates LPS 1.5 mg/kg, IP, and 20 mg/kg AM was supplemented in the diet. Data were presented as mean ± SD. Different superscript letters indicate a significant difference (*p* < 0.05). Image magnification is 100× and scale bar = 200 μm.

The fluorescence detection of ZO-1 and claudin-3 in the ileum tissue on day 5 is shown in Figure 5. The fluorescence intensity of ZO-1 and claudin-3 in the LC-0 mg/kg group was significantly decreased (*p* = 0.0001; *p* = 0.0002, respectively) compared to the NC group. The LC-(20, 40, and 80 mg/kg) and LA-20 mg/kg treatments significantly increased the fluorescence intensity of ZO-1 and claudin-3 as compared to the LC-0 mg/kg (*p* < 0.05). Interestingly, the LC-80 mg/kg and LA-20 mg/kg treatments obviously reversed the LPS-induced decrease in fluorescence signal intensity of ZO-1 and claudin-3 (*p* < 0.0001).

## 4 Discussion

Worldwide, the high mortality rate in the juvenile ostrich population has greatly influenced the ostrich industry. Up to 50% of ostrich chicks all over the world are lost during the first 3 months (33). In Europe, the average mortality of chicks under 4 weeks old exceeds 50% (34). In South Africa, chicks were deceased, with a mortality rate reaching up to 46.7% before 28 days of age, and an extra 30.7% was seen between 28 and 90 days after hatching (35). According to previous reports, intestinal tract diseases and gut microbiota imbalances are the leading causes of death in juvenile ostriches (6, 36). Modulating the gut microbiota has been reported to lower the mortality associated with intestinal tract disorders (36). Common pathogens responsible for enteritis in ostriches include *Escherichia coli*, *Clostridium* spp., *Campylobacter jejuni*, *Pseudomonas aeruginosa*, *Salmonella* spp., and *Klebsiella* spp. (6, 37, 38). Postmortem inspection of deceased individuals revealed widespread inflammation and hemorrhage in the gut mucosa (2). In this current study, CC supplements enhanced growth rate and shifted gut microbiota in growing ostrich chicks. Furthermore, CC attenuated gut inflammation and improved the histological architecture of the gut epithelium. Our current findings appear to be well substantiated by other studies on ostriches (39), growing pigs (20), sheep (21), and broiler chickens (23). The CC supplementation could improve the palatability of the forage-based diet in sheep and subsequently increase their daily dry matter intake and digestibility aptitude (21). Moreover, the mean weight gain observed in the ostriches during the first 4 months of life is in accordance with previous studies (40, 41). Adding CC forage to the cereal diet increased daily feed intake, growth performance, and feed conversion ratio in growing pigs (19) and broiler chickens (42). The highest level of mortality in ostrich chicks has been found to occur in the first month of life (2, 35, 43). Hence, in this current study, the fecal microbiota of one-month-old chicks was sequenced to help identify disparities in the gut microbial composition. Our findings were in line with a prior study (39), showing a positive correlation between ileal microbial diversity and the ostrich’s growth rate. Additionally, the abundance of the phylum *Bacteroidota* was mostly enriched in the ostrich ilea, which mainly ferments the dietary carbohydrate and produces a pool of short chain fatty acids as energy sources (44). Furthermore, CC significantly increased the population levels of the genus *Phascolarctobacterium* and *Phascolarctobacterium succinatutens* spp. while simultaneously decreasing the levels of the family *Clostridiaceae* and the genus *Clostridium*. Beneficial taxa are well known to enhance gut barrier integrity and decrease metabolic endotoxemia (39, 45). *Phascolarctobacterium succinatutens* can effectively utilize succinate, a stress-induced signaling mediator produced by other pathobionts, and in turn decrease gut inflammation (46, 47). Conversely, *Clostridiaceae* showed more conspicuous taxonomic patterns, including a tremendous rise in the genus *Clostridium*. *Clostridium* spp. has been reported to induce extensive intestinal damage, gut inflammation, and enterotoxaemia in ostriches, resulting in high morbidity and mortality (2, 6). Our findings revealed drastically higher microbial α and β diversity with CC supplementation, suggesting that CC could improve the composition of gut microorganisms. The functional microbial species identified in this study coincide with various reports at various taxonomic levels (2, 36, 39). The impact of gut microbiota on health and metabolic processes is widely documented (48), and the variation of the gut microbiota could regulate most of the biological processes in the body, thereby affecting health and growth performance (49). In this current study, the outcome of the KEGG pathway analysis revealed that gut taxonomic compositions associated with the CC supplementation were closely related to functional biosynthesis pathways, such as amino acid biosynthesis and fatty acid and lipid biosynthesis. In agreement with our findings, CC supplementation in growing-finishing pigs could increase the amino acid concentrations, such as threonine, aspartic acid, phenylalanine, histidine, alanine, and glycine (50). Additionally, CC supplementation could improve the systemic lipid profile, as indicated by decreased levels of cholesterol, triglyceride, and low-density lipoprotein levels (50, 51).

The TLR4/NF-κBp65 signaling pathway is a key regulator of intestinal inflammation (52, 53), and also plays a vital role in maintaining gut barrier integrity (54). LPS, a major endotoxin derived from gut gram-negative bacteria, acts on TLR4/NF-κBp65, causing overproduction of pro-inflammatory cytokines that disrupt intestinal TJs and eventually lead to intestinal permeability (55, 56). In this current study, CA markedly downregulated proinflammatory cytokines and subsequently alleviated intestinal inflammation induced by LPS in juvenile ostriches via the inactivating TLR4/NF-κBp65 signaling pathway (Figure 4). The TLR4 transduction cascade has been involved in the regulation of LPS-induced TJ permeability and mucosal damage (55). Managing gut leakage is crucial to improving the health and performance of the birds (57). In a variety of LPS-induced inflammatory models, CA has been evaluated and evidenced for its anti-inflammatory and antioxidant effects in acute liver injury (27), nerve injury (25), and acute lung damage (28). Additionally, CA supplementation enhanced the growth performance of the juvenile ostriches, which could be attributable to the modifications in the intestinal histomorphology with increasing villus height. Inclusion of CC forage in the basal diet resulted in increased villus length in growing pigs (20) and broiler chickens (23). The height of the intestinal villi are positively correlated with absorption surface area, nutrient bioavailability, and better utilization of dietary energy (58, 59). Using natural medicinal plants as potential alternatives to antibiotic growth promoters in animal husbandry provides ubiquitous advantages, such as solving the drug residue issue, as well as improving the quality and quantity of meat production (60). For instance, herbal dietary supplementation was evidenced to improve growth performance and feed conversion ratios in broiler chickens (61, 62) and promote lean mutton quality and production in sheep (63). Our findings revealed that the supplementation of CA at a dose of 80 mg for 5 consecutive days could decrease the gut inflammatory process, improving the ostrich’s growth performance to a level comparable to the effect of AM treatment at a dose of 20 mg/kg. Therefore, our findings prompt antibiotic-free production cycles and open the doors for animal farming to adopt CA as a therapeutic and/or growth promoter substitute.

The current study provides valuable information about CC and CA supplementation, either on the gut microbial diversity or the growth performance of juvenile ostriches. However, the small sample size for sequencing data analysis in this study may have limited the value of observations as a true representation of gut microbial diversity in the ostrich population. In addition, the outdoor access of ostriches was occasionally limited by some inclimental environmental conditions, and the outside temperature and humidity were uncontrollable, which may have had a certain impact on the experiment’s repeatability. In the future, it will be a vital issue for further studies with larger sample sizes to investigate the long-term impact of CC and CA supplementation in a controlled environment. As well, *in vitro* research using the ostrich’s intestinal cell culture model is needed to fully authenticate the effect of CA in ostriches.

## 5 Conclusion

The CC forage supplementation could enhance body weight and lower the incidence of fatal enteritis in juvenile ostriches. These enthralling findings were probably due to pivotal shifts in the gut microbiota by increasing the abundance of *Phascolactobacteria* and decreasing the enrichment of *Clostridium*. Additionally, CA could efficiently mitigate the gut inflammation and maintain intestinal barrier function by suppressing the IL-1β/IL-6/TNF-α-driven inflammatory response and enhancing the expression of ZO-1 and claudin-3 TJ proteins. This current study indicates that the use of CC as a feed supplement might be a potential therapeutic and/or growth promotor alternative or complement to antimicrobials in the animal industry.

## Data Availability

Sequencing data presented in this study can be found in online repository. The name of the repository and accession number can be found below: https://www.ncbi.nlm.nih.gov/sra/PRJNA1082686.

## Footnotes

^1^ www.randomizer.org

## Supplementary material

Supplementary Figure S1 **(A)** The sequence information of 12 fecal samples of both non-chicory (NC) and chicory (CC) groups. **(B)** Venn diagram for bacterial ASV/OTU distribution in the NC and CC fecal samples. **(C)** Rarefaction curves. **(D)** Accumulation curves. **(E)** Rank abundance curve.

Supplementary Figure S2 Significant alternations in the bacterial abundance at different classification levels in both non-chicory (NC) and chicory (CC) groups. All of the data represent means ± SD. *p < 0.05; **p < 0.01; ***p < 0.001, and **** p < 0.0001.

Supplementary Figure S3 Mean proportion (%) of predicted metabolic pathways of ileal bacterial species between non-chicory (NC) and chicory (CC) groups. The significant differences were observed at 95% confidence level and p < 0.05.

Supplementary Table S1 Raw data of weight gain of juvenile ostriches in the chicory (CC) forage supplementation experiment and data of weight gain, intestinal villi length, western blot (WB), and immunofluorescence analysis in the CA supplementation experiment.

Supplementary Data Sheet S1 Original western blot (WB) images generated from the CA supplementation experiment show expressed TLR4/NF-κBp65 pathway-related proteins and tight junction (TJ) proteins in the ileal tissues.
