# Supplementary figures and images for: Chicory supplementation improves growth performance in juvenile ostriches potentially by attenuating enteritis
Source: Front Vet Sci. 2024 Sep 23;11:1432269. doi: 10.3389/fvets.2024.1432269 (PMC11457291; doi:10.3389/fvets.2024.1432269)

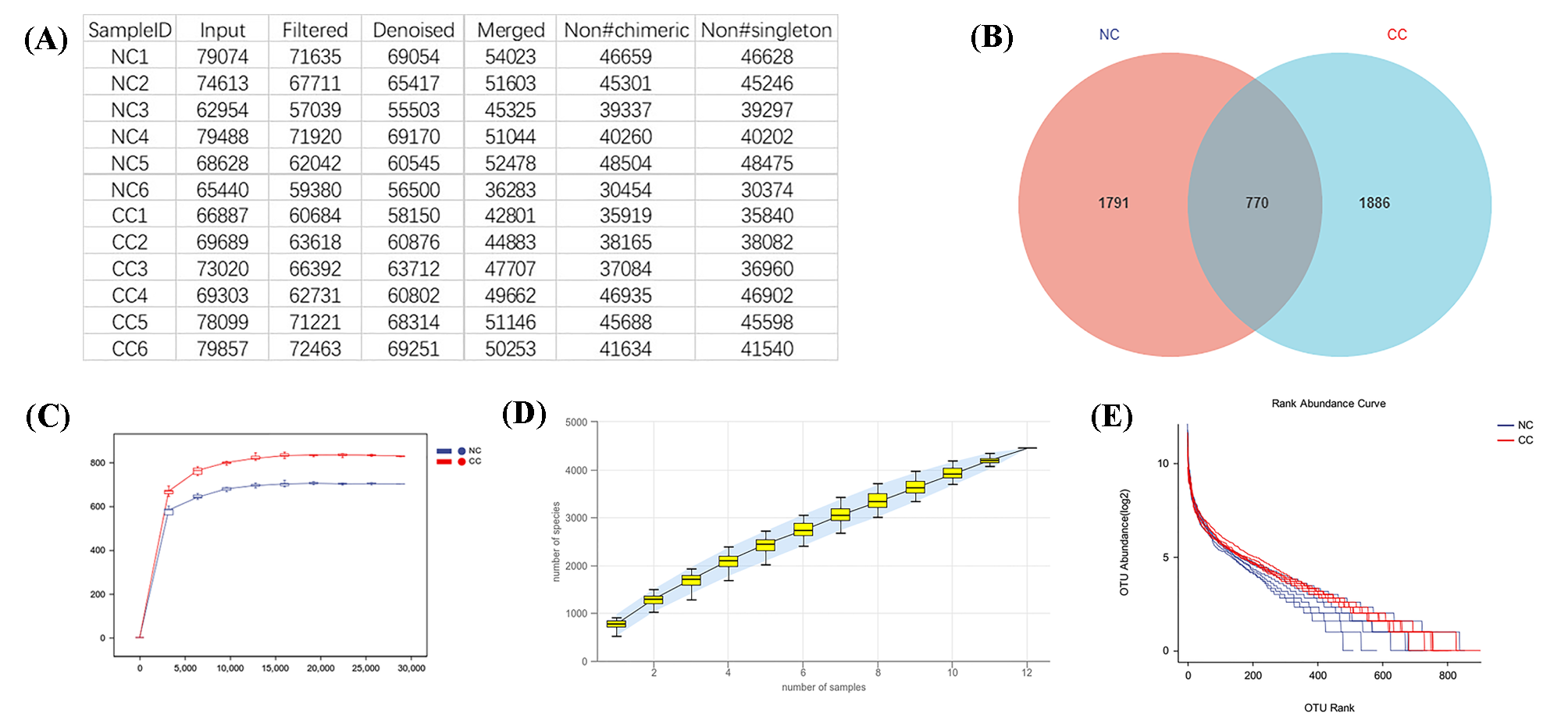

Supplement: Supplementary Figure S1 — (A) The sequence information of 12 fecal samples of both non-chicory (NC) and chicory (CC) groups. (B) Venn diagram for bacterial ASV/OTU distribution in the NC and CC fecal samples. (C) Rarefaction curves. (D) Accumulation curves. (E) Rank abundance curve. [file Image_1.tif]

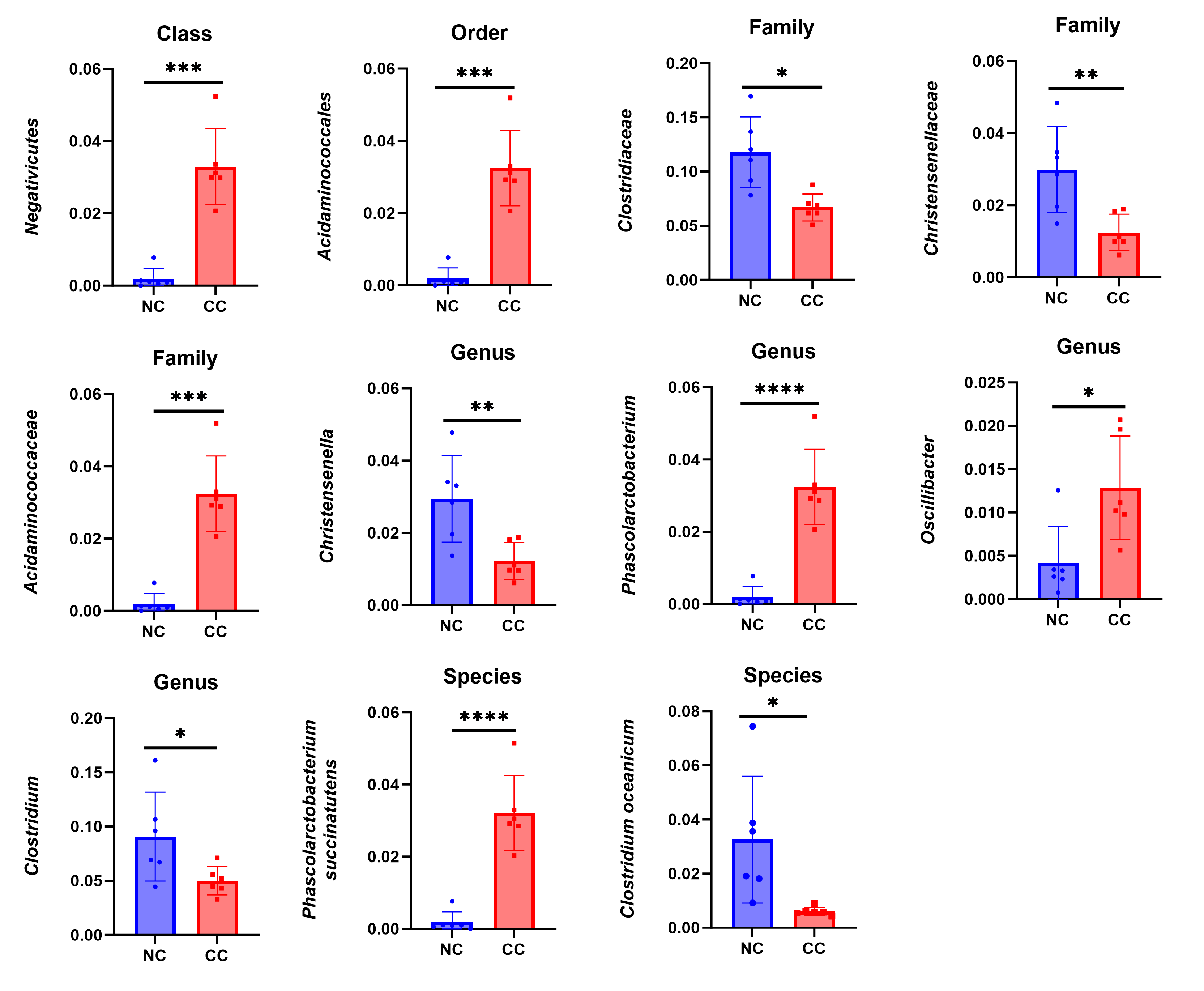

Supplement: Supplementary Figure S2 — Significant alternations in the bacterial abundance at different classification levels in both non-chicory (NC) and chicory (CC) groups. All of the data represent means ± SD. *p < 0.05; **p < 0.01; ***p < 0.001, and **** p < 0.0001. [file Image_2.TIF]

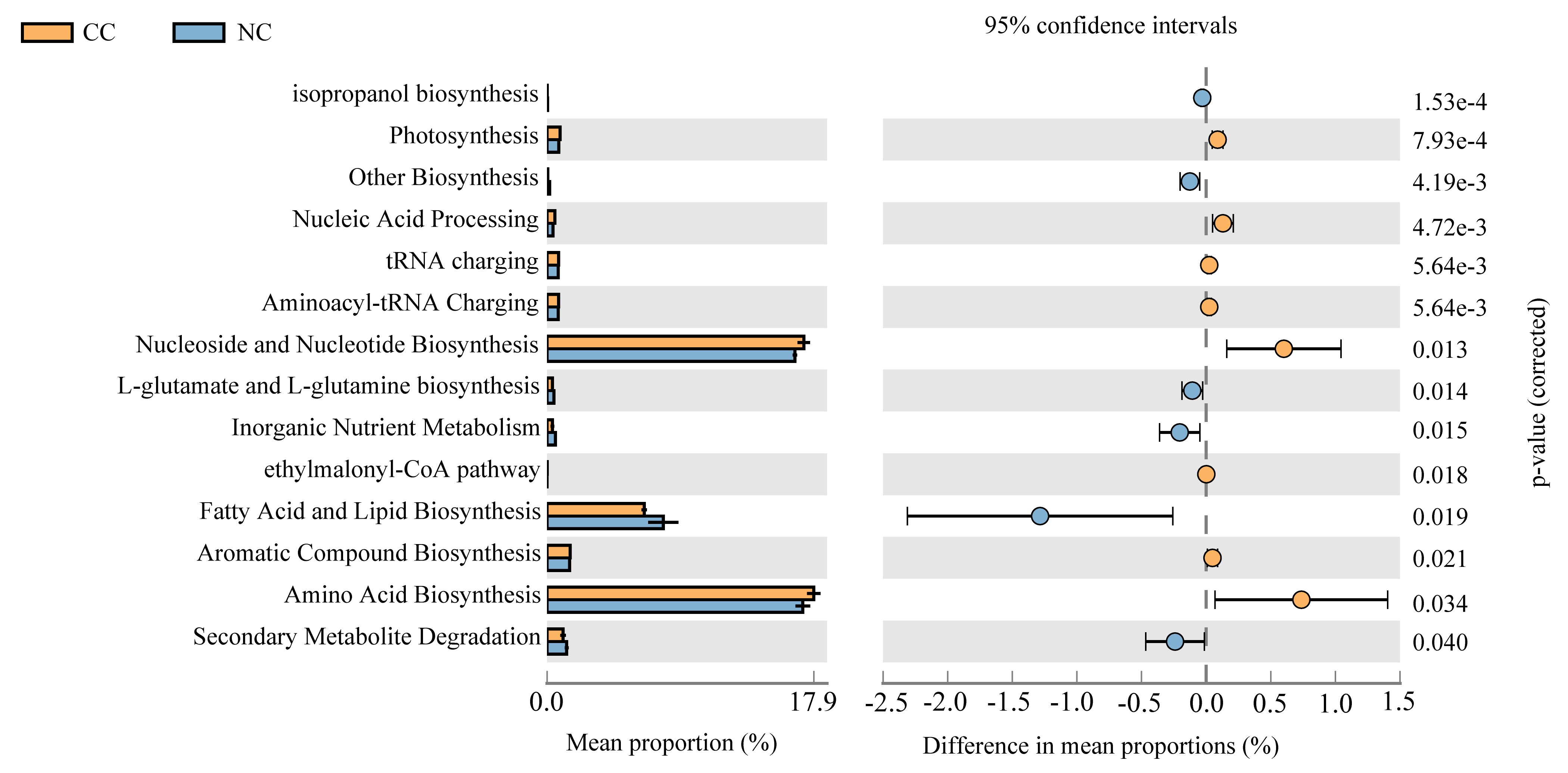

Supplement: Supplementary Figure S3 — Mean proportion (%) of predicted metabolic pathways of ileal bacterial species between non-chicory (NC) and chicory (CC) groups. The significant differences were observed at 95% confidence level and p < 0.05. [file Image_3.JPEG]

**Claudin3 22KD**


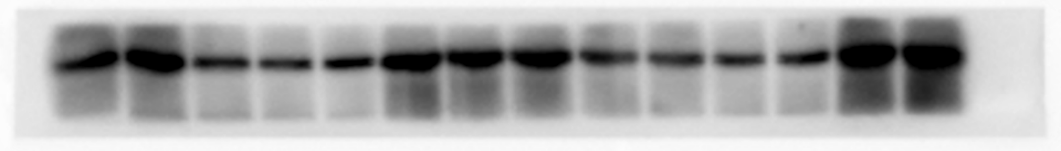

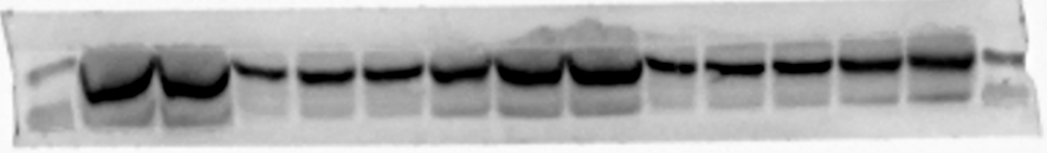


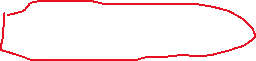

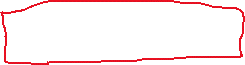


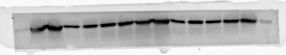


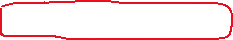


**GAPDH 37KD**


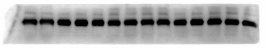


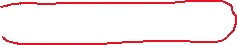

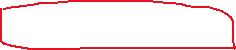


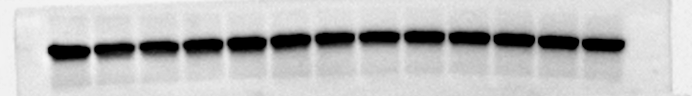

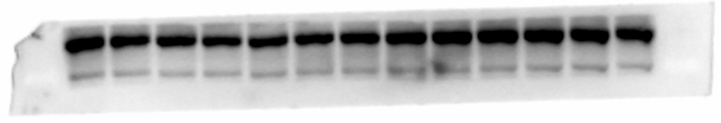


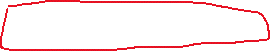

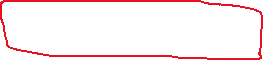




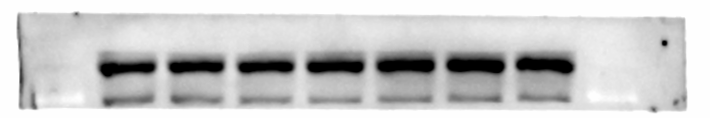


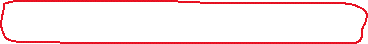

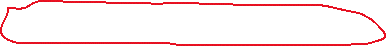





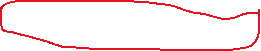


**IL-1β 17KD**


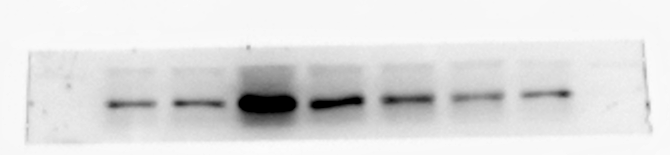


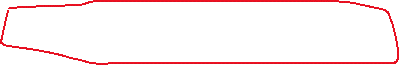


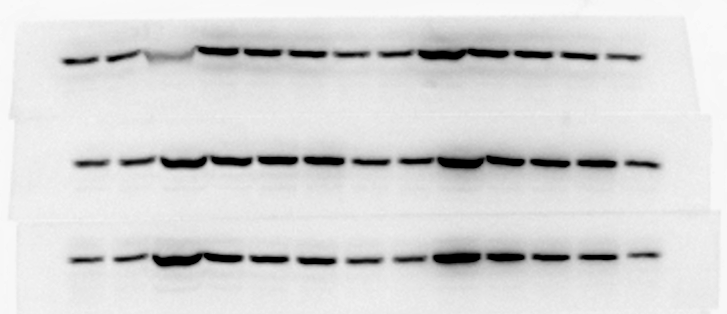


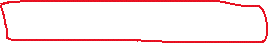

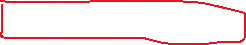

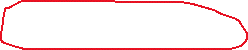


**IL-6 21KD**


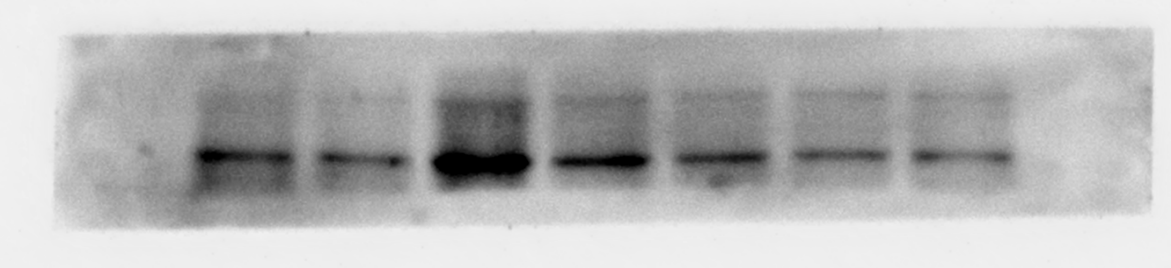


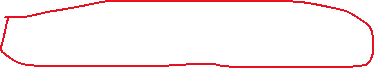


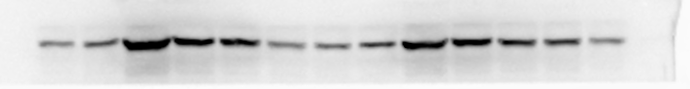


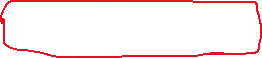


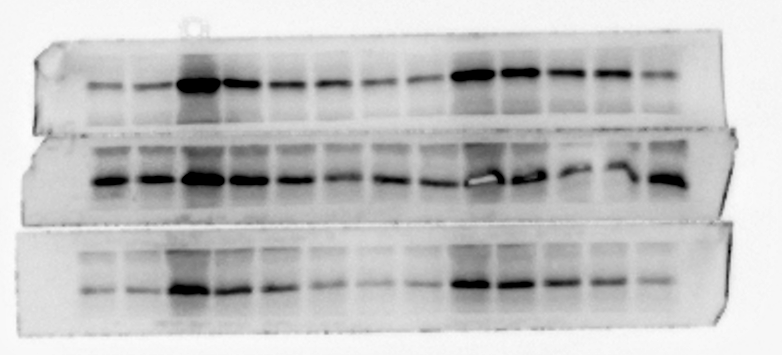


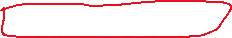

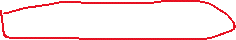


**IL-10 20KD**


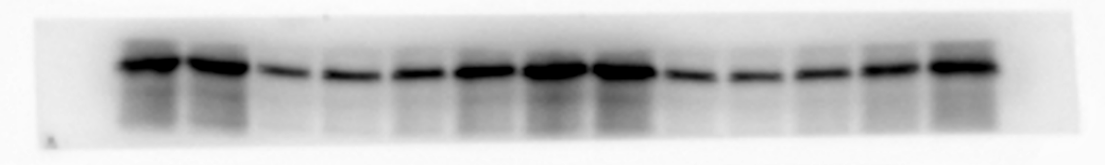


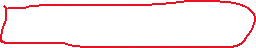


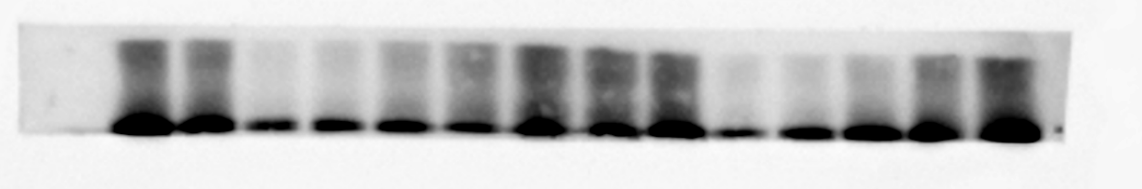


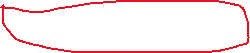

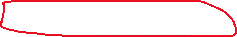


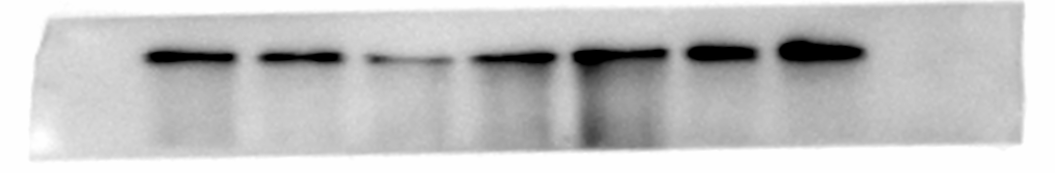

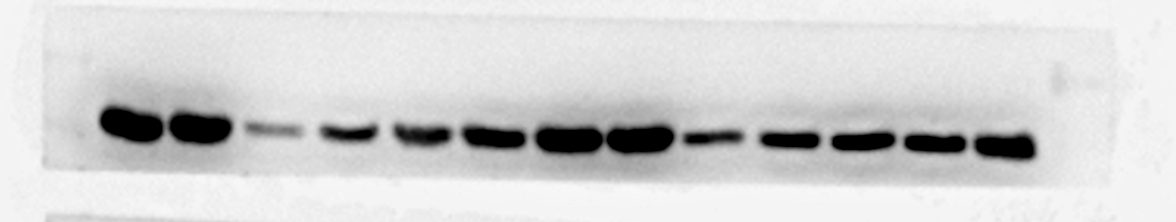


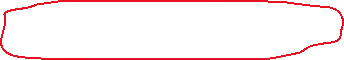

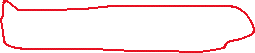


**P65 65KD**


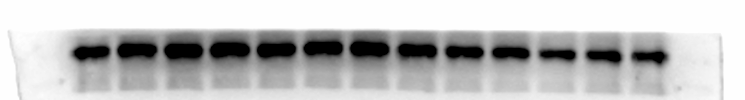


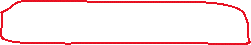


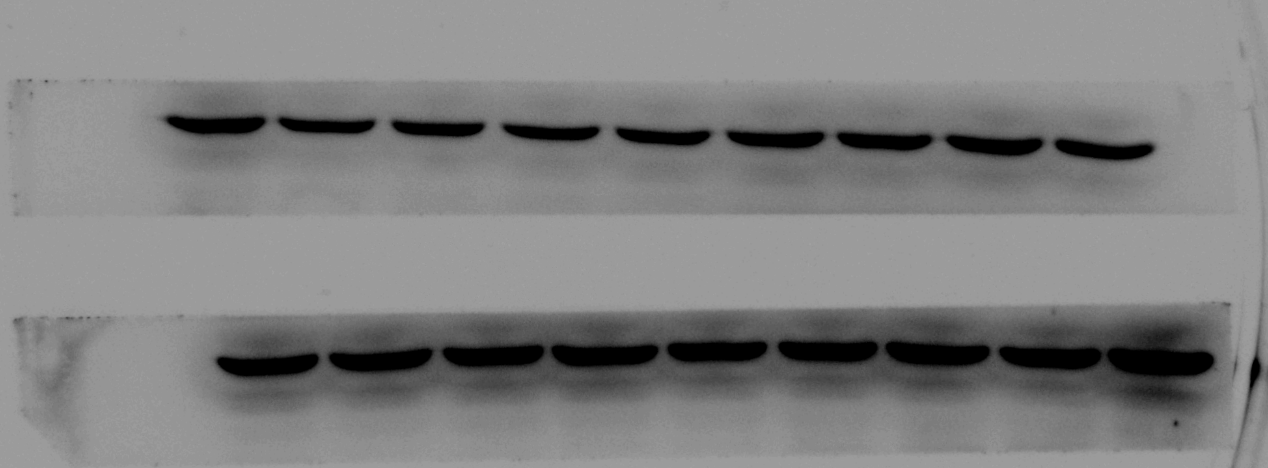


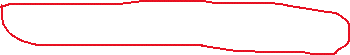

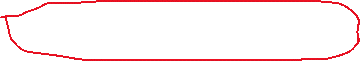


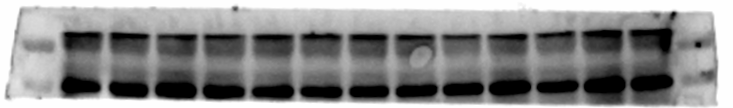


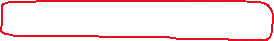


**P-P65 65KD**







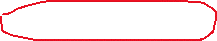

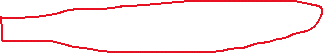


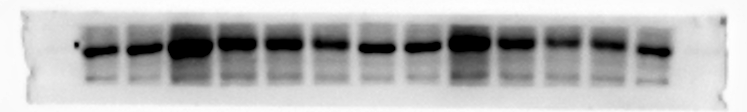


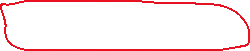


**TGF-β 25KD**


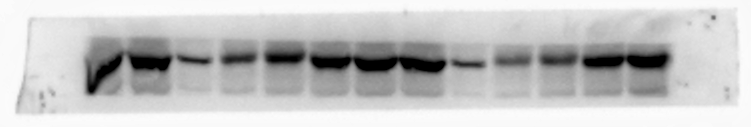


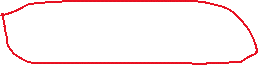


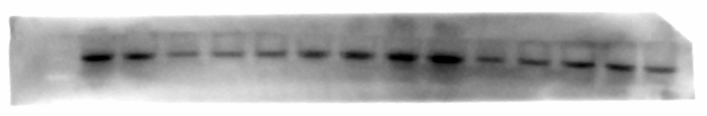


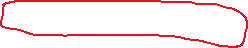

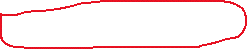


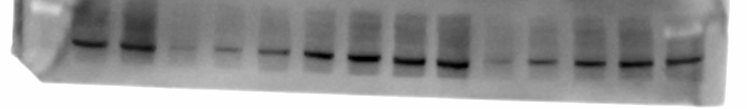


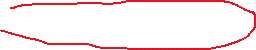

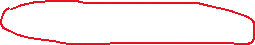


**TLR4 95KD**


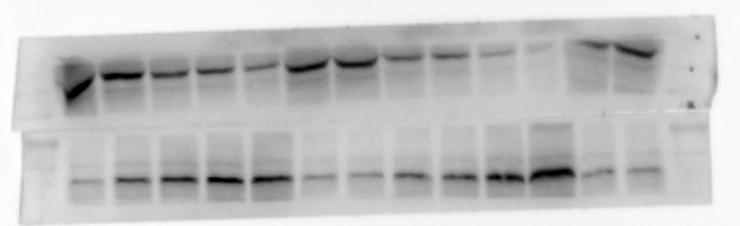

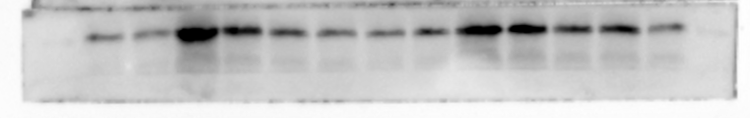

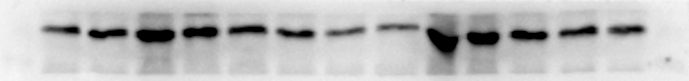


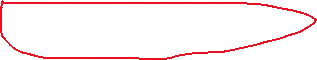

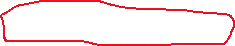

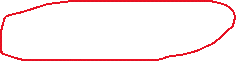

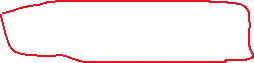


**TNF-α 17KD**


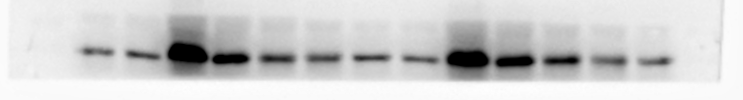


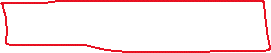


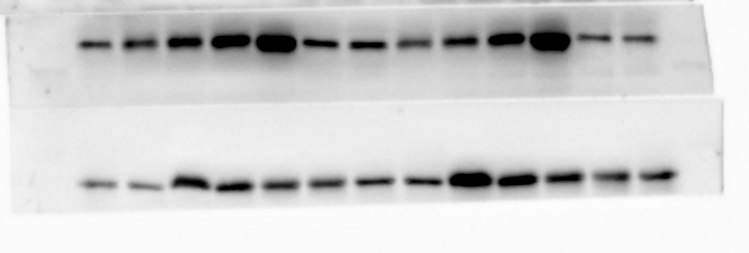


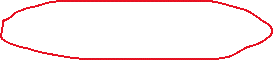

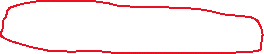


**ZO-1 220KD**


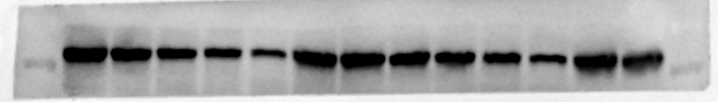


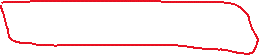


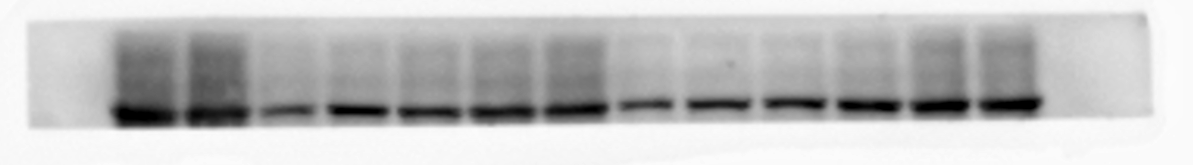


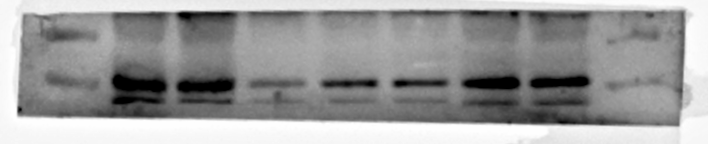

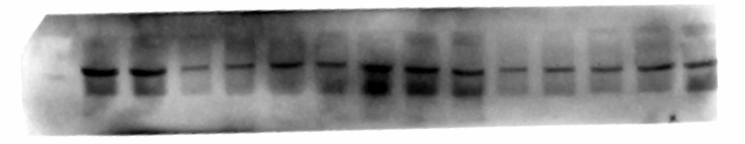


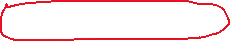

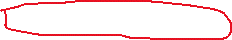

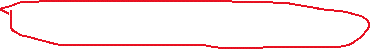

Supplement: Supplementary Data Sheet S1 — Original western blot (WB) images generated from the CA supplementation experiment show expressed TLR4/NF-κBp65 pathway-related proteins and tight junction (TJ) proteins in the ileal tissues. [file Data_Sheet_1.DOCX]
